# Current Insights on Fiber Posts: A Narrative Review of Laboratory and Clinical Studies

**DOI:** 10.3390/dj11100236

**Published:** 2023-10-10

**Authors:** Dayana Campanelli de Morais, Sheila Butler, Maria Jacinta Moraes Coelho Santos

**Affiliations:** Division of Restorative Dentistry, Schulich School of Medicine and Dentistry, Western University, London, ON N6A 5C1, Canada

**Keywords:** nonvital tooth, fiber posts, luting agents, post length

## Abstract

Purpose: The aim of this study was to review the literature related to the clinical performance and laboratory findings regarding fiber posts, as well as the cementation technique employed with their use. Materials and Methods: A literature search was performed using an electronic database, PubMed/Medline, between 2010 and 2023. The terms used were “intra coronal post, fiber post, post cementation, and post length”. Titles and abstracts were initially screened, and a full-text assessment was conducted for those that fulfilled the inclusion criteria. The reference list of the collected papers was also screened for further relevant citations. Results: In this work, 135 potentially eligible studies were analyzed. Titles and abstracts of 90 studies followed the inclusion criteria and were selected for a full-text assessment, resulting in 50 studies selected. Moreover, additional studies from relevant citations were included, totaling 57 studies. Conclusion: According to the laboratory and clinical studies revised, the survival rate between fiber and prefabricated and cast metal posts was similar, and failures were mainly related to the loss of retention. The intra-canal post length of less than two-thirds of the root length presented successful results when ferrule was present. Furthermore, the ferrule increased the longevity of teeth restored with fiber posts. Additionally, the use of a surface treatment protocol for fiber posts and the adhesive cementation technique both contributed to the clinical success and longevity of the intra-canal post.

## 1. Introduction

Endodontically treated teeth commonly present a high risk of biomechanical failure due to the loss of tooth substance and frequently require a prosthetic restoration [1]. The decision for the placement of a post mostly depends on the quantity and the quality of the remaining tooth structure. Several studies related the presence of a ferrule (±2 mm) [2,3,4,5] and the number of remaining walls [5,6,7,8,9,10,11] to the ability of the post and core complex to resist intraoral forces regardless of the type of post and final restoration. Previous in vitro studies [6,7] observed that the fracture resistance of endodontically treated teeth enhanced significantly with the increase in the number of coronal walls. In addition, biomechanical aspects and tooth location have been evaluated in several studies, and it has been shown that anterior teeth are at a higher risk of failing due to the lateral forces they are subjected to during function, whereas posterior teeth are subject primarily to vertical forces [4,12,13,14].

The increasing demand for aesthetics, especially in the anterior region, led to the search for restorative alternatives for metallic post systems, such as fiber posts. The prefabricated fiber post’s microstructure comprises a resin matrix, which is usually made of epoxy resin or its derivatives [15], allowing the post to present an elastic modulus similar to the dentine (fiber post ≅ 30 GPa; dentine ≅ 18 GPa) compared to the metallic post (108.6 GPa) [16]. As a result, the absorption and distribution of stress are more uniform throughout the radicular reminiscent, reducing the risk of nonrestorable fracture [16]. 

Most fiber post systems allow some light transmission through the root canal; however, the light transmission and bonding strength decrease from the cervical to the apical third [17]. Moreover, intraradicular dentine adhesion is still a clinical challenge due to its limited access and visibility associated with a reduced number of dentinal tubules at the apical third due to the presence of irregular secondary dentine among other structures that could be associated with the risk of the system debonding after long periods in the oral environment [18]. 

The selection of posts deserves special attention since their physical and mechanical properties can influence the pattern of stress distribution throughout the tooth. A variety of prefabricated post systems have been introduced, such as cast metallic posts, prefabricated metallic, and more recently, translucent fiber posts [15]. Likewise, a large number of luting agents presenting different compositions and technique protocols are available for cementation [19]. Therefore, the purpose of this review was to summarize the most updated literature from laboratory and clinical studies regarding intra-canal posts in an attempt to provide an updated evidence-based guideline to help clinicians in the selection of the most appropriate system and cementation protocols in dental practice.

## 2. Methods

### 2.1. Eligibility Criteria

The criteria for selecting articles in the present review followed these specific aspects: articles had to be written in English, undergo peer review, and be dated between 2010 and 2023 (Table 1). 

### 2.2. Data Sources

An electronic literature search was conducted using the PubMed search engine combining the keywords “fiber post”, “intra coronal post”, “post cementation”, and “post length”. The studies encompassed prospective and retrospective clinical trials as well as in vitro studies. Furthermore, as part of the review process, the search was extended by reviewing the reference sections of the retrieved articles to gather additional relevant information.

### 2.3. Search Strategy

**Figure 1:**
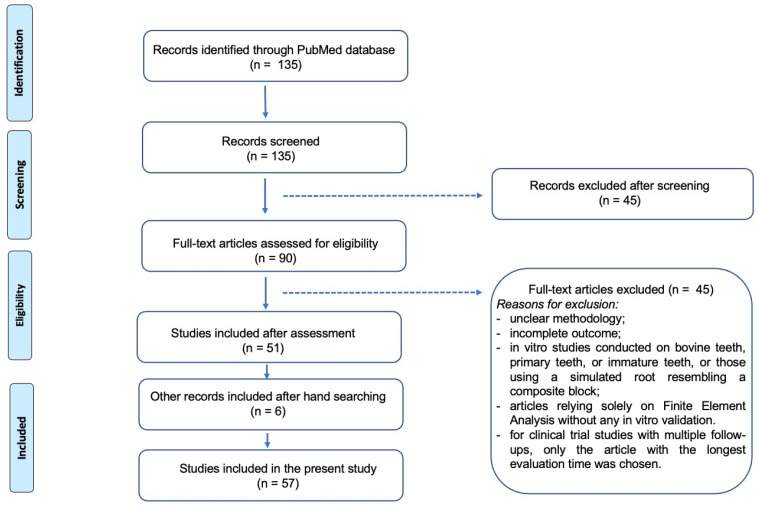
Flow diagram (PRISMA format) of the screening and selection process.

After eliminating duplicate papers, 135 abstracts of potential studies underwent screening, resulting in the selection of 90 articles that focused on fiber posts intra-canal length, cementation protocol, and survival. Exclusion criteria entailed eliminating articles relying solely on finite element analysis without any in vitro validation, in vitro studies conducted on bovine teeth, primary teeth, or immature teeth, or those using a simulated root resembling a composite block. Regarding clinical trial studies, in cases where multiple publications from the same study reported different follow-up periods, the article with the longest evaluation time was selected. Following a thorough examination of the full articles, 51 studies were selected. In addition, the references of the selected papers were screened for further relevant citations, resulting in the inclusion of 6 additional studies, totaling 57 studies (Figure 1).

### 2.4. Data Included

The information extracted from each study, such as authors and year of publication, type of posts, luting agent, and tooth location, is presented in Table 2. Studies that did not include one of these pieces of information were excluded. Data regarding the mechanical properties of the materials used, the influence of remaining dental structure on the outcome, and the survival rate including reasons for failures from clinical and in vitro studies were evaluated and discussed in the present investigation. 

## 3. Results and Discussion

### 3.1. Features of Fiber Posts

The use of prefabricated posts with core buildups has shown several advantages, such as the use of a direct and less time-consuming technique [20]. Specifically, nonmetallic prefabricated post systems have shown high fracture resistance due to their ability to bond to the tooth structure and capacity to better absorb and distribute stress compared to the metallic posts [21]. This fact has been claimed to provide a short-term strengthening effect called an endodontic “Monoblock” [22].

Several in vitro studies have compared the use of posts with different elastic modulus, such as zirconia [23], carbon fiber [24,25], stainless steel [25], prefabricated titanium [26], glass fiber, and cast posts [13,16,23,24,25,26,27,28], and verified that posts with high elastic modulus transmit stress to the root tip resulting in root fracture, while posts with a dentin-like modulus are able to distribute occlusal stresses more evenly in the root canal, reducing the risk of fractures. 

On the other hand, the most frequent failures of teeth restored with fiber posts have been related to post/core debonding [4,26,27,28,29], which, most often, have resulted in favorable fractures likely to be restored. In regard to the mechanical properties (Table 1), different results have been published. While several laboratory studies have verified higher fracture resistance [20,23,30] and bond strength to the dental structure [31] of glass fiber posts compared to different types of posts, other studies have observed similar bond strength and fracture resistance when comparing prefabricated metal and cast posts with fiber posts [13,25,31,38,39,40]. Furthermore, some studies reported lower bond strength and fracture resistance values for glass fiber posts compared to metal posts [21,24,26,27,28,32,33,34]. It is essential to note that the maximum bite force for posterior teeth has been reported to range from 420 ± 112 N to 632 ± 174 N [48]. Interestingly, this range coincides with the fracture resistance of most available fiber post systems.

### 3.2. Length of Fiber Posts

Conventionally, it has been established that the intra-canal post length should respect two-thirds of the total length of the remnant dental structure, or at least the height of the crown, or no less than half of the alveolar bone height surrounding the root to provide sufficient retention and avoid catastrophic fracture of the root walls [1]. Also, the post size that best fits the dowel space will be chosen, respecting the amount of dental structure remaining [6,7,13]. Besides these aspects, several other factors should be analyzed when placing an intra-canal post, including root length, crown height, level of bone support, and ferrule [15].

Due to the features of fiber posts, such as better stress distribution and a more favorable fracture mode, several in vitro studies [11,15,25,28,34,35] have suggested the use of fiber posts with short intra-canal length (one-third of the root length ≥ 6 mm). A study from Thakur A and Ramarao S [20] verified that fiber posts cemented just above half of the root length (or about 8 mm long) presented mechanical behavior statistically similar to longer posts cemented at two-thirds of the root length. 

Although decreasing the post length presents the advantages of preserving dentin structure, reducing the risk of root perforation [25,35], and allowing re-intervention on the canal when needed, short posts may reduce the intra-canal retention [1]. On the other hand, an increased intra-canal post length may not provide increased retention of fiber posts cemented with resin luting agents since several investigations [38,40,41,42,43] have reported a decreased quality of bonding at the apical area due to both anatomic features and technical difficulties. Additionally, some studies [2,15,34] found no significant difference among the intra-canal post lengths analyzed. They reported that unless the intra-canal post length is less than one-third of the root length, the result may not significantly influence the fracture resistance of the treated teeth, considering that the correct cementation protocol was followed [2].

### 3.3. Cementation

The use of resin luting agents has been preferred for post cementation, due to satisfactory retention and resistance against post fracture [35]. Some factors related to the anatomical and histological characteristics of the root canal are likely to influence the adhesion of luting cement, resulting in a variation of dentin bonding among different areas of the same tooth [42]. Regarding the types of adhesive systems available, although the etch-and-rinse mode has been traditionally used with resin cement [10,14,49,50], the advances in the adhesive systems resulted in simplified adhesive protocols through the use of self-etch adhesives and self-adhesive resin cement, allowing to shorten chair time and simplify the clinical procedures. Additionally, the use of self-adhesive resin cement avoids the difficult task of applying and rinsing the phosphoric acid in the apical area of the prepared canal, resulting in a more predictable and less technique-sensitive procedure [37,51].

Although a variety of materials and protocols for post cementation have been extensively analyzed, in vitro investigations have provided contradictory findings. Several studies have shown significantly higher bond strength values (Table 1) for self-adhesive luting cement [37,38,39,41,42,43,44,45,51,52], while others have shown comparable behaviors among the adhesive systems available [22,46]. In fact, it has been reported that the composition of the self-adhesive resin cement favors a positive performance, due to a greater moisture tolerance than self-etching cement for instance [38,42]. Contrary to these findings, other studies have reported a less favorable bond strength and fracture resistance of self-adhesive cement compared to etch-and-rinse adhesives and self-etching adhesive cement [36]. The large variation in results may be related to several factors including the variation in teeth’s anatomy, different preparation and pretreatment utilized. These factors may play an important role during the cementation protocol [46]. 

Furthermore, it has been mentioned that the longevity of the cementation procedure can be influenced by the type of bonding generated between the luting material and the tooth structure [9,41,51]. Additionally, their unique compositions can affect water sorption and subsequent hygroscopic expansion of the cement [19,41]. 

### 3.4. Surface Treatment of Fiber Posts

Regarding the surface treatment of fiber posts, different protocols have been tested. Among them, etching the post with phosphoric acid (36% phosphoric acid for 15 s) and/or coating the post with a silane primer [37] and/or with an adhesive bonding agent [47] have been the most common approaches. The silanization of fiber posts has been considered advantageous to improve their retention into the root canals depending on the post type [37,46]. Previous studies that have evaluated the use of silane on titanium [4] and fiber posts observed no debonding after an average follow-up of 8.8 years [50] and after a follow-up of 11 years [4]. In addition, a micro-mechanical post-surface pretreatment consisting of airborne-particle abrasion has been reported to significantly improve retention due to the roughness created, which contributed to an increased surface area and surface energy [47,53]. Furthermore, the idea of customizing glass fiber posts has also been mentioned in the literature. It is claimed that a thicker layer of adhesive cement may lead to unfavorable stress distribution at the post-cement–dentine interface and reduce post retention [35].

### 3.5. Survival from Longitudinal Studies

Since oral conditions are dynamic, long-term clinical evaluation studies provide the most reliable evidence. Some studies have reported encouraging results on the clinical longevity of fiber posts [3,4,49] with a survival time of over 10 years. Several investigations that evaluated prefabricated metal and fiber posts [54,55,56] observed similar survival rates for both types of retainers. 

A long-term randomized clinical trial [4] evaluated glass fiber and titanium posts (Fiberpoints Root Pins Titanium) luted with a self-adhesive cement (RelyX Unicem; 3M ESPE). A 2 mm ferrule and post length of 9 mm were employed. The root canal and tooth surface were cleaned with an air abrasion system, and the post space was rinsed with 2 mL of 99.6% ethanol solution and dried with paper points. The titanium posts were treated with a tribochemical silica coating (2.8 bar, 13 s, Rocatec Soft, 3M ESPE), and a thin layer of silane (ESPE-SIL; 3M ESPE) was applied and air-dried in both types of posts. The authors reported that the survival rate decreased rapidly after the 5-year evaluation as survival rates decreased from 86.4% to 58.7% and from 92.5% to 74.2% for fiber glass and titanium posts, respectively, at the 5- and 8-year follow-ups. In addition, the most common failure mode was horizontal root fracture, followed by endodontic failure with apical periodontitis. 

A study by Naumann et al. (2012) [49] evaluated two types of fiber posts, consisting of a parallel-sided post with serrated surface configuration (FibreKor, Jeneric Pentron) and a tapered post shape (Luscent Anchors, Dentatus), both in the sizes of 1.0, 1.25, and 1.5 mm. The posts were luted with an etch-and-rinse adhesive system (EBS-Multi, 3M ESPE, Germany) with a 10-year follow-up. Posts were cleaned with alcohol, air-dried, and coated with a thin layer of bonding agent. In spite of the fact that the authors observed a 2-fold increased failure rate for anterior teeth compared to premolars and molars, there was no significant difference among the fiber posts used. Other studies have also verified a higher number of failures for anterior teeth [3,49,54]. Contrary to these findings, some investigations reported more failures in the posterior region [14]. This finding might be attributed to the fact that only 72 teeth were analyzed and the short 3-year evaluation period since posterior teeth are predisposed mostly to vertical forces while anterior teeth are predisposed to lateral forces. Therefore, anterior teeth are at a greater risk of failing [4,12,13,14].

A randomized clinical study [10] investigated the use of a prefabricated glass post (DT Light Post) and a customized glass FRC post (everStick Post). Both systems were luted to the root canal using resin cement (Calibra and BisCore) to restore premolars with single crowns. The authors reported an overall survival rate of 94.1% after 6 years of evaluation with higher effectiveness for the prefabricated fiber posts compared to the custom-made posts. A significantly increased success rate was observed on teeth with an increased number of remaining walls.

Another randomized clinical trial [14] compared the survival of glass fiber (White Post DC, FGM) and cast metal (CoCr) posts used to restore teeth with no remaining coronal walls. Posts were inserted in two-thirds of the root canal and restored with single crowns. The filling was removed from the root canal with #5 Gates Glidden burs (Dentsply Maillefer). Fiber posts were cleaned with ethanol, pretreated with silane (ProSil, FGM), and luted with two types of resin cement (RelyX ARC, 3M ESPE) and self-adhesive resin cement (RelyX U100, 3M ESPE). After up to 3 years of follow-up, no significant difference was observed between the types of cement and types of post, and survival rates of 97.1% (*n* = 35) for fiber posts and 91.9% (*n* = 37) for cast metal posts were observed. A higher survival rate was verified for anterior teeth (97.5%) compared to posterior teeth (90%). 

In addition, a randomized clinical trial [54] compared the clinical outcomes of a prefabricated glass fiber post (Parapost FibreLux, Coltène-Whaledent, Cuyahoga Falls, OH, USA), a custom-made glass fiber post (everStick, StickTech—GC America, Alsip, IL, USA), and a gold cast post and core (gold-alloy-based wrought—Parapost, Coltene-Whaledent, and Medior 3 Cendres + Métaux) luted with self-etching adhesive cement (Panavia F 2.0/ED Primer II, Kuraray). After 5 years of follow-up, the authors reported an overall survival rate of 91.4% for fiber posts, 92.1% for custom-made glass fiber posts, and 91.2% for gold cast post and core. The most common type of failure observed was dislodgement of posts, consisting of 30.9% for anterior teeth and 18.02% for posterior teeth, and mostly with custom-made glass fiber posts (everStick, StickTech). Furthermore, a previous investigation [57] evaluated the effectiveness of a quartz translucent fiber post (DT Light SL9; VDW GhB, Munich, Germany) assessed at a 2-year evaluation. Posts were silanized and cemented with self-etching cement (Calibra, Dentsply, Kostanz, Germany). The authors reported an excellent clinical performance of the fiber posts with no periapical lesions. 

A longitudinal retrospective study [50] analyzed endodontically treated teeth with and without posts after an average period of 8.8 years (Easy Post and Easy Post Lux, Dentsply). The initial post-space preparation was performed with a Largo Peeso Bur and precision drills. The post was placed in teeth possessing only one wall and/or less than one-third of the remaining height of the clinical crown. The post length used was 8 mm, and they were cleaned with alcohol and coated with silane (Monobond S/Monobond Plus; Ivoclar Vivadent, Schaan, Lichtenstein). Posts were luted with resin cement using an etch-and-rinse technique. The survival rate of teeth restored with fiber post (94.3%) was significantly higher than the teeth without a post (76.3%), irrespective of the restoration type, and no post debonding was observed. The authors concluded that teeth with only one remaining wall restored with fiber posts yielded significantly less tooth loss and reported that the use of crowns did not improve the prognosis when compared with noncrowded teeth. 

## 4. Final Remarks/Considerations

Limitations of this review can be attributed to the limited number of longer-term clinical trials published in this area and studies lacking precise clinical guidelines for different clinical situations. Therefore, long-term evidence from clinical studies with better-defined guidelines is needed. Furthermore, this review paper aimed to search for the most updated papers (from 2010 and onward) available on the PubMed search engine, which may have constrained the search. However, the primary objective of this review was to provide current insights related to fiber posts and cementation protocol, with a focus on reviewing the most up-to-date literature over the last 13 years. 

Based on the papers evaluated in the present review, a similar survival rate was observed for fiber and prefabricated metal posts, as well as cast metal, which ranged from 3 to 10 years. The lower modulus of elasticity of fiber posts was associated with favorable outcomes. The primary causes of failures observed in fiber posts were related to post/core debonding. Moreover, employing a surface treatment protocol, like silane, was demonstrated to enhance adhesion for most types of fiber posts. Both conventional and self-adhesive cement contributed equally to the clinical success and longevity of intra-canal posts. 

Furthermore, the presence of a ferrule was deemed crucial for the longevity of the post/restoration complex. Under these conditions, intra-canal fiber post lengths of less than two-thirds of the root length can be effectively utilized. Future research should delve into areas that have not been addressed in this review, such as the optimal canal irrigation and cleansing methods prior to adhesive cementation, protocols for gutta-percha removal, and techniques for extracting fiber posts.

## Figures and Tables

**Table 1:** Inclusion and exclusion criteria.

Inclusion Criteria	Exclusion Criteria
English language	Before 2010
Studies focused on fiber posts intra-canal length; cementation protocol; survival studies	Finite element analyses paper without in vitro validation; meta-analyses; systematic review; literature review
Prospective and retrospective clinical trials and in vitro studies	In vitro studies conducted on bovine teeth; primary teeth or immature teeth
	In vitro study which used a simulated root resembling a composite block

**Table 2:** Results from in vitro studies.

Authors and Year	Types of Posts	Length	Mechanical Properties		Luting Agent	Teeth Localization
**Fracture Resistance (N)**						
Amarnath et al., 2015 [1]	Stainless steel (Parapost, Coltene Whaledent)	4 mm	122 N (7.11 N)		Paracore dual cure resin cement, Coltene Whaledent, Cuyahoga Falls, OH, USA	Mandibular premolars
		7 mm	246 N (6.81 N)			
		10 mm	188.5 N (5.74 N)			
	Glass fiber (Parapost Fiber-lux, Coltene Whaledent)	4 mm	68.5 N (7.11 N)			
		7 mm	137.5 N (6.81 N)			
		10 mm	140.5 (5.74)			
Mobilio et al., 2013 [2]	Glass fiber post (GFP; Size #2 RelyX Fiber Post, 3M ESPE AG, Espepl, Seefeld, Germany)	5 mm	1736.4 (1113.8)		Self-adhesive resin cement (RelyX Unicem Aplicap, 3M ESPE, Espepl, Seefeld, Germany)	Mandibular premolars
		7 mm	1038.6 (600.2)			
Zicari et al., 2013 [5]	Without fiber post/ RelyX Posts (3M-ESPE, Espepl, Seefeld, Germany)	10 mm	Without ferrule	361.5 (151.7)/ 577.0 (104.9)	Panavia F 2.0 (Kuraray, Tokyo, Japan)	Upper premolars
			392.51 N (76.30 N)/388.00 N (71.97 N)	758.5 (121.9)/ 647.6 (132.9)		
Hou, Gao and Sun 2013 [6]	Without fiber post	8 mm	Coronal walls 0	850 (120)	Self-etch; Bisco Inc., Schaumburg, IL, USA	Single-rooted mandibular premolars
			1	1020 (170)		
			2	1680 (220)		
			3	1940 (450)		
			4	1980 (300)		
	Quartz fiber posts (D.T. Light-Post; Bisco Inc., Schaumburg, IL, USA)		0	1410 (360)		
			1	1580 (180)		
			2	2070 (390)		
			3	2160 (370)		
			4	2210 (430)		
Mangold and Kern 2011 [7]	Glass fiber posts (Komet ER DentinPost; ISO size 90, Brasseler) were airborne-particle abraded for 5 s at a distance of 30 mm with 50 µm alumina particles (Heraeus Kulzer) at 0.25 MPa Without fiber post	7.5 mm	0	537.6 (55.1)	Panavia 21 TC; Kuraray Medical Inc., Tokyo, Japan)	Mandibular premolars
			1	672.3 (7.5)		
			2	756.8 (126.8)		
			3	1065.9 (211.8)		
	Without fiber post		0	335.6 (39.7)		
			1	497.2 (93.5)		
			2	702.4 (95.9)		
			3	885.3 (208.8)		
Valdivia et al., 2012 [8]	Intact teeth	10 mm	844.8 (186.5)		Self-adhesive resin cement (RelyX Unicem 2; 3M ESPE)	Maxillary central incisors
	Class III with prefabricated glass fiber post (Exacto Translucido No. 3; Angelus Science and Technology, Londrina, PR, Brazil)		894.1 (397.4)			
Torres-Sánchez et al., 2013 [9]	Glass fiber posts (Tenax; Coltène/Whaledent, Altstätten, Switzerland)/ Type IV gold alloy (Argendent 90; The Argen Corp, San Diego, CA, USA)	10 mm	127.91 (14.02)/ 79.92 (5.66)		RelyX Luting; 3M ESPE, St. Paul, Minn	Single-rooted premolars
			48.21 (4.61)/ 38.04 (3.89)		RelyX ARC; 3M ESPE	
			39.04 (3.78)/ 55.40 (5.88)		Multilink System Pack; Ivoclar Vivadent, Schaan, Liechtenstein	
Ramírez-Sebastià et al., 2014 [11]	FRC Postec Plus (Ivoclar Vivadent, Schaan, Liechtenstein)	10 mm	432.6 (55.3)		Clearfil DC Bond, Kuraray, Japan	Upper central incisors
		5 mm	470.9 (55.2)			
Castro et al., 2012 [13]	Exacto (Angelus, Londrina, PR, Brazil)—glass fiber post: cleaned with 70% alcohol and a silane agent was applied (Angelus, Londrina, PR, Brazil)	2/3rd	655.6 (145.8)/ 2940.5 (917.3)/ 2217.8 (691.1)/ 2854.2 (642.9)		RelyX-U100 (3M ESPE, Seefeld, Germany)	Maxillary incisors/maxillary canines/maxillary two-rooted premolars/mandibular first molars (with ferrule)
	Kromalit (Knebel, Porto Alegre, RS, Brazil)—Ni–Cr alloy post and core: sandblasted with aluminum oxide particles (50 μm) under 2 bars pressure for 10 s and cleaned in distilled water		711.3 (154.7)/ 3278.6 (702.5)/ 2161.4 (602.2)/ 2934.0 (785.9)			
Remo et al., 2010 [15]	Quartz fiber posts (Endo Light post) Glass fiber posts (White Post DC #2, FGM, Joinville, SC, Brazil)	5 mm	41.68 (5.31)		Dual cured resin cement (Prime&Bond NT + Fluorocore 2)	Single-rooted premolars
		7 mm	44.88 (6.77)			
		9 mm	510 (199.8)			
Barcellos et al., 2013 [16]	Fiberglass posts (Angelus, Londrina, PR, Brazil) covered with resin composite Z250 (B0.5, Z250, 3M ESPE)	9 mm	260.23 (69.74)		Rely X ARC (3M ESPE)	Upper canine teeth
	Nickel–chrome alloy (Ni–Cr alloy, Kromalit; Knebel, Porto Alegre, RS, Brazil)		241.35 (68.27)			
Thakur and Ramarao 2019 [20]	Custom-made glass fiber (Angelus Rua Goias, Londrina, PR, Brazil)	1/2th/ 2/3rd	159.97 (34.06)/ 166.84 (33.11)		Luxa core Z-dual-cure (DMG, Hamburg, Germany)	Mandibular first premolars (without ferrule)
	Prefabricated glass fiber (Reforpost, Angelus, Londrina, PR, Brazil)		224.2 (32.9)/ 250.33 (15.40)			
	Prefabricated carbon fiber (Reforpost, Angelus, Londrina, PR, Brazil)		204.07 (29.63)/ 201.39 (41.44)			
	Ribbond (Ribbond Inc., Seattle, WA, USA)—polyethylene fiber post		146.44 (13.53)/ 179.75 (33.52)			
Li et al., 2011 [21]	D.T. Light FRC post (Bisco Inc., Schaumburg, IL, USA)	10 mm	305.73 (76.34)		Conventional glass ionomer cement (Fuji, GC Corp., Tokyo, Japan)	Maxillary central incisors (without ferrule)
	Macro-Lock FRC post (RTD Inc., Saint-Egrève, France)		449.50 (113.18)		ParmaCem (DMG Inc., Hamburg, Germany)	
	Ni–Cr alloy cast post (Bego, Bremen, Germany)		511.09 (91.95)			
Gopal et al., 2017 [22]	EasyPostTM (Dentsply Maillefer) Whitepost DC (FGM)—glass fiber: abraded by airborne particles for 5 s using 50 μm alumina particles at 0.1 MPa	10 mm	657.80 (57.37)		Calibra Esthetic (Dentsply Maillefer)—etch and rinse	Maxillary central incisors (without ferrule)
			762.40 (251.49)		PermaFlo DC (Ultradent Pord. Inc., South Jordan, UT, USA)—self-etch	
			258.3 (12.7)		SmartCem (Dentsply Maillefer)—self-adhesive	
Habibzadeh et al., 2017 [23]	Ni–Cr alloy (Wiron 99, Bego, Bremen, Germany	2/3rd	780.59 (270.53)		Panavia F2.0 (Kuraray, Noritake, Dental Inc., Tokyo, Japan)	Premolars (with ferrule)
	Zirconia post and core using MAD-MAM (Zirkonzahn, Gais, Italy)		435.34 (220.41)			
	Light post (Illusion X-RO, RTD, Saint Egreve, France)—glass fiber		915.71 (323.60)			
Solomon and Osman 2011 [24]	Luscent Anchors (Dentatus, NY, USA)—glass fiber post	8 mm	678.84 (199.45)		Parapost cement (Coltene Whaledent, West Sussex, UK)	Maxillary incisor (with ferrule)
	Parapost fiber White (Coltene, Whaledent, Mahwah, NJ, USA)—carbon fiber post		653.01 (208.86)			
	Surtex Classic Posts (Dentatus, NY, USA)—titanium post		682.82 (208.86)			
	Custom cast dowel and core (nickel–chromium alloy)		1673.41 (490.74)			
Chuang et al., 2010 [25]	Carbon fiber post (J. Morita, Osaka, Japan)	10 mm/ 5 mm	1248.81 (117.60)/ 1253.76 (79.68)		Bistite II DC (Tokuyama Dental Corp., Tokyo, Japan)	Maxillary anterior teeth (with ferrule)
	Glass fiber post (J. Morita, Osaka, Japan)		1292.33 (185.86)/ 1247.17 (53.03)			
	Stainless steel post (J. Morita, Osaka, Japan)		973.27 (115.42)/ 1338.79 (121.84)			
Maroulakos, Nagy, and Kontogiorgos 2015 [26]	Parapost XH (Coltene/Whaledent)—titanium alloy	11 mm	123.5 (23.4)		Panavia 21 (Kuraray Noritake Dental Inc., Tokyo, Japan)	Anterior maxillary teeth (without ferrule)
	D.T. Light-Post (Bisco Inc.)—quartz fibers		117.6 (19.3)			
	Ney-Oro 60 (Dentsply Intl)—gold alloy		174.0 (51.0)			
Ok et al., 2014 [27]	Cast post core	2/3rd	1949.35 (316.0)		Bifix, QM, (Voco GmbH, Cuxhaven, Germany)	Maxillary canine teeth
	Glass fiber post (Uicore Ultradent, Salt Lake City, UT, USA)		1722.48 (144)		Rebilda (Voko, Cuxhaven, Germany)	
	Glass fiber post (Uicore Ultradent, Salt Lake City, UT, USA)		1486.19 (191.7)		Bifix, QM, (Voco GmbH, Cuxhaven, Germany)	
Franco et al., 2014 [28]	Fibrekor (Jeneric/Pentron)—fiber glass post: cleaned with 70% ethanol and water, and silanized (Cleafil SE Bond Primer, Kuraray, Co., Ltd., Kuraray Medical Inc., Tokyo, Japan)	10 mm/ 7.5 mm/ 5 mm	236.08 (19.68)/ 212.17 (17.12)/ 200.01 (28.07)		Panavia 21 (Kuraray, Osaka, Japan)	Maxillary canines (without ferrule)
	Type IV gold alloy (Stabilor G; Degussa Dental AG)—cast post and core (control)	10 mm	634.94 (53.2)			
Doshi et al., 2019 [29]	Glass fiber posts (Coltene Whaledent, OH, USA)	10 mm	343.89 (10.44)		Rely X Ultimate adhesive universal resin cement (3M ESPE, St. Paul, MN, USA)	Maxillary central incisors
	everStick Post (GC, Europe) foil		452.32 (14.35)			
	Carbon fiber posts (Angelus, Londrina, Brazil)		281.26 (10.81)			
	Without fiber post		576.52 (20.39)			
Jindal et al., 2012 [30]	Ribbond (Ribbond Inc. Seattle, WA, USA)—polyethylene fiber post	10 mm/ 5 mm	216.930 (53.40)/ 299.62 (53.42)		Monocem (Shofu dental)	Maxillary central incisors (with ferrule)
	Glass fiber post (Fibrapost No.2, Produits Dentaires S.A., Vevey, Switzerland)		740.21 (29.87)/ 425.18 (42.73)			
Kivanç, Alaçam, and Görgül 2010 [31]	everStick Post (Stick Tech Ltd., Turku, Finland)—custom-made glass FRC post	10 mm	936.58 (299.83)		Panavia F (Kuraray)	Single-rooted maxillary premolars
	Filpost (Filhol Dental, Baltimore, MD, USA)—titanium post		891.50 (243.17)			
	Polyethylene woven fiber post (Ribbond Inc., Seattle, WA, USA)		827.25 (275.52)			
	Without post		920.33 (162.24)			
Rippe et al., 2014 [32]	Ni–Cr alloy (Wironia Light, Bego, Bremen, Bremen, Germany)	10 mm	707.5 (125.6)		Self-adhesive resin cement (RelyX U100, 3M ESPE, St. Paul, MN, USA)	Single-rooted teeth
	Glass fiber posts (White Post DC #2, FGM, Joinville, SC, Brazil)		510 (199.8)			
Mastrogianni et al., 2021 [33]	Glass fiber posts (FRC Postec Plus, Ivoclar Vivadent)	9 mm	1422.85 (344.11)		Panavia F 2.0, Kuraray, Tokyo, Japan	Mandibular premolars
	Prefabricated metal posts (Stainless steel, Parapost, Coltene)		2427.17 (497.96)			
	Without post		224.36 (196.25)			
Palepwad and Kulkarni 2020 [34]	Cast metal post	6 mm	269.02 (88.22)		Dual-polymerizing resin cement (LuxaCore Z)	Central incisors
		8 mm	299.15 (92.13)			
	Glass fiber post (Hi-Rem post)	6 mm	143.03 (49.17)			
		8 mm	178.18 (56)			
	Zirconia post (ER Cera post)	6 mm	216.91 (66.43)			
		8 mm	299.70 (113.95)			
Zicari et al., 2012 [35]	RelyX Posts (3M-ESPE, Seefeld Germany) Without fiber post	10 mm	392.51 (76.30)/ 388.00 (71.97)		Panavia F 2.0 (Kuraray, Tokyo, Japan)/ RelyX Unicem (3M-ESPE, Seefeld, Germany)	Upper premolars
		7.5 mm	404.81 (149.77)/ 443.96 (166.23)			
		5 mm	440.52 (222.31)/ 499.20 (189.76)			
Samran et al., 2018 [36]	Whitepost DC (FGM)—glass fiber (Angelus, Londrina, PR, Brazil)	10 mm	258.3 (12.7)		RelyX Ultimate Clicker (3M ESPE, St. Paul, MN, USA)—etch and rinse	Mandibular first premolars (with ferrule)
			218.7 (11.1)		Breez (Pentron, Orange, CA, USA)—self-adhesive	
			165.4 (8.9)		Ketac Cem (3M ESPE, St. Paul, MN, USA)	
**Bond strength (MPa)**						
Reis et al., 2011 [19]	Glass fiber posts (Fibrekor, Jeneric Pentron Incorporated, Wallingford, CT, USA)	9 mm	7.66 (2.67)		Self-cured resin cement C&B Cement (Bisco, Schaumburg, IL, USA	Single-rooted teeth
			7.16 (4.29)		Glass ionomer cement Ketac Cem (3M ESPE, St. Paul, MN, USA)	
			2.80 (1.04		Resin-modified glass ionomer cement GC FujiCEM (GC Corp., Tokyo, Japan)	
Farina et al., 2011 [37]	Fiberglass posts (Angelus, Londrina, PR, Brazil)	2/3rd	8.11 (2.30)/ 3.28 (0.82)		RelyX-Unicem (3M ESPE, Seefeld, Germany)/ Cement-post (Angelus, Londrina, PR, Brazil)	Maxillary canines (without ferrule)
	Carbon fiber posts (Angelus, Londrina, PR, Brazil)		5.13 (1.34)/ 2.27 (0.074)			
da Silva et al., 2015 [38]	Exacto post (Angelus, Londrina, Brazil)	10 mm	14.32 (2.84)/ 11.56 (4.13)		Breeze self-adhesive (Pentron Clinical Tec, Wallingford)/ Panavia F 2.0 (Kuraray, Osaka, Japan)	Single-rooted teeth (without ferrule)
	everStick Post (StickTeck Ltd., Turku, Finland)		16.89 (2.66)/ 13.69 (3.26)			
Yaman et al., 2014 [39]	Glass fiber (radix; dentsply-maillefer)	10 mm	13.9 (3.8)/ 9.9 (2.9)		Panavia F 2.0 (Kuraray, Osaka, Japan)/ RelyX Unicem (3M ESPE)	Single-rooted premolars
	Zirconia post (B&L Biotech Co., Fairfax, VA, USA)		7.2 (2.1)/ 11.5 (4.0)			
Başaran et al., 2019 [40]	Snow post (Carbotech, Ganges, France)—zirconia glass fiber	10 mm	9.3 (2.3.80)		Duo-link (Bisco, Schaumburg, III)	Maxillary central incisors (with ferrule)
	Ribbond (Ribbond Inc., Seattle, WA, USA)—polyethylene fiber		8.24 (1.89)			
	D.T. light-post (Bisco, Schaumburg, III)—quartz glass fiber		8.87 (3.08)			
	Cytec blanco (Hahnenkratt, Konigsbach-Stein, Germany)—glass fiber		9.2 (2.78)			
Pereira et al., 2013 [41]	Reforpost N.2 (Angelus, Londrina, PR, Brazil)	10 mm	19.1 (7.4)		CG Gold label (GC Corp., Tokyo, Japan)	Maxillary canines (without ferrule)
			9.6 (7.2)		Rely X ARC (3M ESPE, Paul, MN, USA)	
			16.4 (3.4)		BisCem (Bisco, Schaumburg, IL, USA)	
			14.0 (3.6)		RelyX U100 (3MESPE, Paul, MN, USA)	
Onay, Korkmaz, and Kiremitci 2010 [42]	Whitepost DC (Whitepost DC 1; FGM)—glass fiber	10 mm	15.41 (1.54)		All Bond SE/Duo-Link (Bisco, Inc., Schaumburg, IL, USA)	Incisors (without ferrule)
			12.6 (1.9)		All Bond 3/Duo Link (Bisco, Inc., Schaumburg, IL, USA)	
			16.13 (1.94)		BisCem (Bisco, Inc., Schaumburg, IL, USA)	
			10.7 (1.68)		Clearfil ED primer II/Clearfil Esthetic Cement (Kuraray)	
Özcan et al., 2013 [43]	Snowpost (Kuraray, Tokyo, Japan)	10 mm	22.4 (2.46)		RelyX Unicem (3M ESPE)	Mandibular premolars (with ferrule)
			19.8 (2.46)		Panavia F 2.0 (Kuraray, Osaka, Japan)	
			18.1 (2.45)		Maxcem (Kerr, West Collins Orange, CA, USA)	
			23.8 (2.5)		Clearfill SA (Kuraray, Osaka, Japan)	
Bitter et al., 2012 [44]	RelyX Fiber Post size 2 (3M ESPE)	8 mm	13.2 (9.5)		Panavia F 2.0 (Kuraray, Osaka, Japan)	Maxillary central (without ferrule)
			13.2 (10.6)		Variolink II (Ivoclar Vivadent, Schaan, Liechtenstein)	
			18.3 (10.3)		RelyX Unicem (3M ESPE, Seefeld, Germany)	
Leme et al., 2011 [45]	DC White Post (FGM)—glass fiber post	10 mm	3.81 (1.07)		RelyX ARC (3M ESPE, Paul, MN, USA)	Single-rooted teeth (without ferrule)
			4.26 (2.29)		RelyX Unicem (3M ESPE)	
Jongsma et al., 2010 [46]	D.T Light-Post (RTD, St. Egreve, France)	12 mm	4.8 (1.9)		Clearfil DC Core (Kuraray)—etch and rinse	Canines (without ferrule)
			5.4 (3.2)		RelyX Unicem (3M ESPE)—self-adhesive	
			4.9 (3.4)		Panavia F 2.0 (Kuraray)—self-etching	
Albashaireh et al., 2010 [47]	Fiber glass post (EasyPost; Dentsply Maillefer) with no treatment/ Acidic treatment (36% phosphoric acid for 15 s)/ Airborne-particle-abrasion treatment (50 mm alumina particles—Heraeus Kulzer GmbH at 2.5-bar pressure, 36.3 psi for 5 s)	10 mm	272.2 (64.6)/ 284.8 (67.1)/ 342.8 (70.3)		Calibra (Dentsply DeTrey)	Maxillary anterior and premolar teeth

## Data Availability

No further data were created.
